# Interleukin-35 modulates the balance between viral specific CD4^+^CD25^+^CD127^dim/-^ regulatory T cells and T helper 17 cells in chronic hepatitis B virus infection

**DOI:** 10.1186/s12985-019-1158-0

**Published:** 2019-04-16

**Authors:** Lanlan Yang, Shengnan Jia, Xue Shao, Siqi Liu, Qian Zhang, Jie Song, Wudong Wang, Zhenjing Jin

**Affiliations:** Department of Hepatopancreatobiliary Medicine, The Second Hospital, Jilin University, No. 218 Ziqiang Street, Nanguan District, Changchun, 130041 Jilin Province China

**Keywords:** Interleukin-35, Regulatory T cells, T helper 17 cells, Hepatitis B virus

## Abstract

**Background:**

Interleukin (IL)-35 regulates imbalance between regulatory T cells (Tregs) and T helper (Th) 17 cells, leading to an important modulator in autoimmune disorder, cancer, and infectious diseases. Our previous study revealed an immunosuppressive activity of IL-35 in chronic hepatitis B virus (HBV) infection. Thus, the aim of the current study was to investigate the role of regulatory function of IL-35 to viral specific Tregs/Th17 cells balance in chronic HBV infection.

**Methods:**

A total of 44 HLA-A2 restricted chronic HBV infected patients, including 21 of chronic hepatitis B (CHB) and 23 of asymptomatic HBV carriers (ASC) were enrolled. Purified CD4^+^ T cells or CD4^+^CD25^+^CD127^dim/−^ Tregs were stimulated with recombinant IL-35. HBV core antigen specific Tregs and Th17 cells were determined by flow cytometry. FoxP3 and RORγt mRNA was measured by real-time PCR. Cytokines production (IL-10 and IL-17) was investigated by ELISA.

**Results:**

Peripheral viral specific Tregs was comparable between CHB and ASC. However, increased percentage of viral specific Th17 cells was found in CHB, leading to the reduction of Tregs/Th17 ratio in CHB patients. IL-35 stimulation elevated viral specific Tregs, but not Th17 cells frequency, in both CHB and ASC, resulting in the elevation of Tregs/Th17 ratio in both groups. This process was accompanied by increased expression of FoxP3 mRNA and IL-10 production, and decreased IL-17 secretion and STAT3 phosphorylation in purified CD4^+^ T cells. Moreover, IL-35 stimulation inhibited viral specific Th17-like phenotype differentiation from Tregs in CHB patients. Effective anti-HBV therapy did not affect viral specific Tregs/Th17 cells frequency or IL-35 expression in CHB patients, however, reduced responsiveness of CD4^+^ T cells or Tregs to IL-35 stimulation in vitro.

**Conclusion:**

Our findings indicated that IL-35 regulation to viral specific Tregs/Th17 balance may contribute to viral persistence in chronic HBV infection.

## Background

Chronic hepatitis B virus (HBV) infection is still a large public health problem, with approximate 350 million persistent infections all over the world [1]. Chronic HBV infection always leads to end-stage liver diseases, such as decompensated cirrhosis, severe hepatitis, and hepatocellular carcinoma (HCC), resulting in more than 1 million deaths annually worldwide [2]. It is well accepted that the outcome of hepatitis B patients is closely related to the interaction between host immune system and virus itself [3]. Acute HBV infection in adults always induces multi-specific T cell-responses, which is pivotal for viral clearance and controlling the infection [4]. However, HBV infection in infants or children always results in persistent infections, which manifested as weak or undetectable cellular immune responses [5]. More importantly, the precise mechanism of T cell hyporesponsiveness and immune tolerance in chronic HBV infection is still not fully understood.

Naïve CD4^+^ T cells can differentiate into different T helper (Th) cells upon activation by various transcriptional factors and cytokines. CD4^+^CD25^+^ regulatory T cells (Tregs) express high level of FoxP3 but low to absent level of CD127 [6], and contribute to immune tolerance via direct cell contact and inhibitory cytokines [mainly interleukin (IL)-10 and IL-35] production during chronic viral infections [7]. Th17 cells express high level of transcriptional factor retinoic acid-related orphan receptor γt (RORγt) and secrete IL-17 and IL-22 [8, 9], which induce inflammation and fibrosis in HBV infection [10, 11]. The imbalance between Tregs and Th17 cells was shown to be associated with liver injury [12] and be an indicator of liver cirrhosis process and a risk factor for HCC occurrence in patients with chronic hepatitis B (CHB) [13]. However, most of the previous reports focused on natural or non-specific Tregs/Th17 cells in chronic viral infection. Few studies revealed HBV-specific Tregs and Th17 cells imbalance during chronic HBV infection.

IL-35, which was mainly secreted by Tregs, is a newly identified IL-12 cytokine family member, and comprises two heterdimeric subunits, IL-12 α chain p35 (IL-12p35) and IL-27 β chain Epstein-Barr virus-induced gene 3 (EBI3) [14, 15]. Our previous studies indicated that elevated serum IL-35 played an immunosuppressive activity and promoted inhibitory function of CD4^+^CD25^+^CD127^dim/−^ Tregs in chronic HBV and hepatitis C virus (HCV) infections [16, 17]. A recent study by Huang et al. showed that IL-35 modulated the imbalance between Tregs and Th17 cells in enterovirus 71-induced hand, foot, and mouth disease [18]. Thus, we hypothesized that IL-35 could also regulate viral specific Tregs/Th17 imbalance in chronic HBV infection. To test this possibility, we investigated circulating HBV-specific Tregs and Th17 cell population and assessed the effect of recombinant IL-35 on HBV-specific Tregs/Th17 function in patients with chronic HBV infection.

## Methods

### Enrolled patients

The study conformed to the ethical guidelines of the 1975 Declaration of Helsinki. The protocol was approved by the Ethics Committee of The Second Hospital of Jilin University on March 2017, and written informed consent was obtained from each participant. Twenty-one of HLA-A2 restricted CHB patients and 23 of HLA-A2 restricted asymptomatic HBV carriers (ASC) were enrolled in the present study. All patients were hospitalized or followed-up in Department of Hepatopancreatobiliary Medicine Affiliated to The Second Hospital of Jilin University between July 2017 and March 2018, and diagnose was made in accordance with diagnostic standard of Chinese Guideline of Prevention and Treatment for Chronic Hepatitis B (2010 Version). All patients were positive for HBV e antigen (HBeAg), and treatment-naïve to nucleos(t)ide analogue and interferon-α. Serum alanine aminotransferase (ALT) presented more than 2-fold elevation in CHB patients, while ALT level was normal in ASC group. No patients received immunomodulatory therapies 1 year before sampling. Patients who were co-infected with other hepatovirus or human immunodeficiency virus (HIV), or afflicted with immune disorder or malignance diseases were excluded from this study. The baseline characteristics of all enrolled patients were shown in Table 1.

**Table 1:** Baseline characteristics of enrolled patients

	Chronic hepatitis B	Asymptomatic HBV carriers
Case (n)	21	23
Gender (Male/Female)	17/4	18/5
Age (years)	29 [18, 42]	25 [19, 37]
Alanine aminotransferase (IU/L)	112.7 [90.4, 356.8]	19.8 [8.9, 31.7]
HBV DNA (log_10_ IU/mL)	5.57 [3.81, 8.13]	6.09 [4.45, 7.97]
HBsAg positive	21	23
Anti-HBs positive	0	0
HBeAg positive	21	23
Anti-HBe positive	0	0
Anti-HBc positive	21	23

Data were presented as median [Q1, Q3]

### Virological and biochemical assessments

HBV DNA was quantified by a commercial real-time polymerase chain reaction (PCR)-Fluorescence Quantitative Detection Kit for HBV DNA (Da’An Gene, Guangzhou, Guangdong Province, China) with detection limit of 2 log_10_ IU/mL. HBV surface antigen (HBsAg), anti-HBs, HBeAg, anti-HBe, and anti-HBV core (anti-HBc) was measured by commercial enzyme linked immunosorbent assay (ELISA) kits (Kehua Biotech, Shanghai, China). Serum biochemical assessments were measured by Hitachi 7500 automatic analyzer (Hitachi, Tokyo, Japan).

### Isolation of peripheral blood mononuclear cells (PBMC) and purification of CD4^+^ T cells/CD4^+^CD25^+^CD127^dim/-^ Tregs

EDTA anticoagulant peripheral bloods were collected from each patients. PBMCs were isolated by density gradient centrifugation using Ficoll-Hypaque (Sigma-Aldrich, St Louis, MO, USA). CD4^+^ T cells or CD4^+^CD25^+^CD127^dim/-^ Tregs were purified from PBMC using CD4^+^ Cell Isolation Kit (Miltenyi, Bergisch Gladbach, Germany) or CD4^+^CD25^+^CD127^dim/-^ Regulatory T Cell Isolation Kit II (Miltenyi) according to manufacturer’s instruction, respectively. The purity of enriched cells was more than 95% by flow cytometry determination.

### Cell culture

CD4^+^ T cells or purified CD4^+^CD25^+^CD127^dim/−^ Tregs were stimulated with recombinant human IL-35 (final concentration: 1 ng/mL; Peprotech, Rocky Hill, NJ, USA) for 12 h. Cells and supernatants were harvested for further experiments.

### Flow cytometry

PBMCs were stimulated with HBV core 18–27 epitope (HBc 18–27, sequence: FLPSDFFPSV; final concentration: 10 μg/mL) in the presence of Brefeldin A (final concentration: 10 μg/mL) for 12 h. Cells were harvested and transferred to FACS tubes, and were stained with anti-CD4-PerCP (BD Bioscience, San Jose, CA, USA), anti-CD25-APC (BD Bioscience), and anti-CD127-FITC (BD Bioscience) for 20 min in the dark at 4 °C. Cells were washed twice with Staining Buffer (BD Bioscience), and were resuspended with 250 μL of Fixation/Permeabilization solution (BD Bioscience) for 20 min at 4 °C. Cells were then washed twice in 1 × BD Perm/Wash buffer (BD Bioscience), and were stained with anti-IL-17-PE (BD Bioscience) for 30 min in the dark at 4 °C. In certain experiments, purified CD4^+^CD25^+^CD127^dim/−^ Tregs were stained with anti-CCR4-FITC (BD Bioscience) and anti-CCR6-PE (BD Bioscience) for 20 min in the dark at 4 °C. Isotype antibodies were used to separate positive and negative cells in PerCP, APC, FITC, and PE fluorescence channels. Samples were analyzed with FACS Calibur analyzer (BD Bioscience). Acquisitions were performed with CellQuest Pro Software (BD Bioscience), and analyses were performed with FlowJo Version 8.4.2 for Windows (Tree Star, Ashland, OR, USA).

### Real-time PCR

Total RNA was isolated using Trizol reagent (Invitrogen, Thermo Fisher Scientific, Carlsbad, CA, USA) according to manufacturer’s instruction. cDNA was synthesized with oligo(dT) primer and random 6 mers using PrimeScript RT Master Mix (TaKaRa, Beijing, China). Real-time PCR was performed using TB Green Premix *Ex Taq* II (TaKaRa). The relative gene expression was quantified by 2^*-ΔΔCT*^ method using ABI7500 System Sequence Detection Software (Applied Biosystems, Foster, CA, USA). The sequences of primers were as following. FoxP3 sense: 5′-CCT CCC CCA TCA TAT CCT TT-3′; FoxP3 anti-sense: 5′-TTG GGG TTT GTG TTG AGT GA-3′; RORγt sense: 5′-AGT CGG AAG GCA AGA TCA GA-3′; RORγt anti-sense: 5′-CAA GAG AGG TTC TGG GCA AG-3′; IL-12p35 sense: 5′-TTC CCA TGC CTT CAC CAC TC-3′; IL-12p35 anti-sense: 5′-TAA ACA GGC CTC CAC TGT GC-3′; EBI3 sense: 5′-TTA CAA GCG TCA GGG AGC TG-3′; EBI3 anti-sense: 5′-TTC CCC GTA GTC TGT GAG GT-3′; GAPDH sense: 5′-GCA CCG TCA AGG CTG AGA AC-3′; GAPDH anti-sense: 5′-TGG TGA AGA CGC CAG TGG A-3′.

### ELISA

Cytokine production in the supernatants were measured using commercial ELISA kits (CusaBio, Wuhan, Hubei Province, China) according to manufacturer’s instruction.

### Western blot

Western blot was performed as described previously [16]. Briefly, cells were lysed on ice for 5 min in 2 × SDS buffer with β-mercaptoethanol, and were treated in 95 °C for 10 min. Supernatants were harvested by centrifugation for 1 min at 10,000×*g*. Total proteins were loaded and separated on SDS-PAGE gels, and were electroblotted onto PVDF membrane. The membrane was soaked for 2 h in block solution, and then incubated overnight in the presence of rabbit polyclonal to signal transducers and activators of transcription 3 (STAT3, phospho Y705, ab76315), or STAT3 (ab32500) (Abcam, Cambridge, MA, USA; 1: 1000 dilution). Horseradish peroxidase-conjugated goat anti-rabbit antibody IgG (Abcam; 1: 2000 dilution) was added for additional 2 h incubation. Antigen-antibody complexes were observed by enhanced chemiluminescence (Western Blotting Luminol Reagent, Cell Signaling Technology, Danvers, MA, USA).

### Statistical analyses

All data were analyzed using SPSS Version 21.0 for Windows (Chicago, IL, USA). Shapiro-Wilk test was used for normal distribution assay. Variables following normal distribution were presented as mean ± standard deviation, and statistical significance was determined by Student *t* test or paired *t* test. Variables following skewed distribution were presented as median [Q1, Q3], and statistical significance was determined by Mann-Whitney test or Wilcoxon matched pairs test. All tests were two-tailed, and *P* value of less than 0.05 was considered to indicate significant difference.

## Results

### Imbalance between HBV core-specific CD4^+^CD25^+^CD127^dim/-^ Tregs and Th17 cells in CHB and ASC

**Fig. 1:**
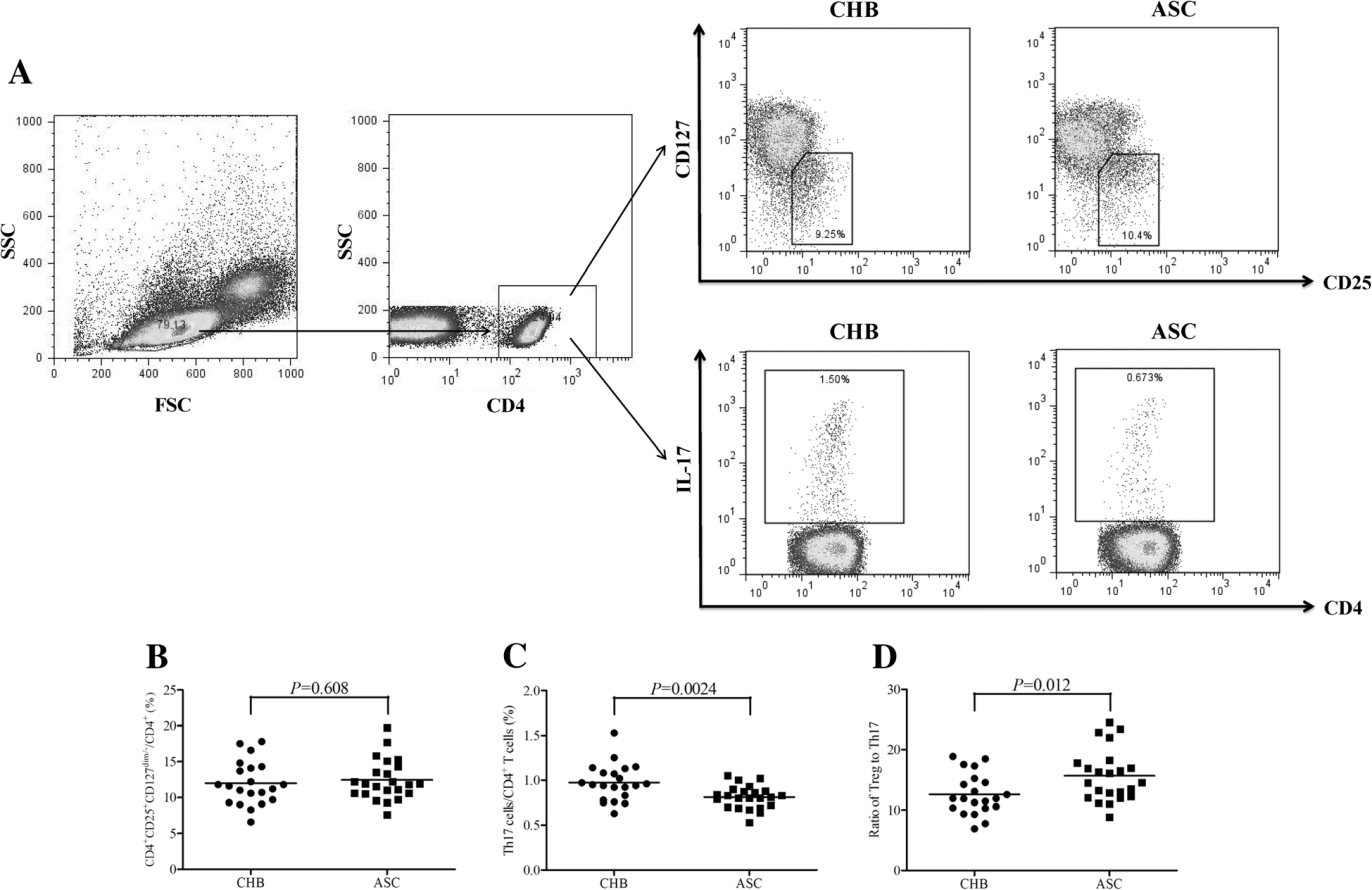
HBV core-specific CD4^+^CD25^+^CD127^dim/−^ regulatory T cells (Tregs) and T helper 17 (Th17) cells in chronic hepatitis B (CHB) and asymptomatic HBV carriers (ASC). Peripheral blood mononuclear cells (PBMCs) were isolated from all enrolled HLA-A2 restricted chronic HBV infected patients (including 23 of CHB and 21 of ASC), and were stimulated with HBc 18–27 peptide for 12 h. Cells were then stained with anti-CD4, −CD25, −CD127, and -interleukin (IL)-17 for flow cytometry analysis. **a** Gating strategy and representative flow dots for CD4^+^CD25^+^CD127^dim/−^ Tregs and CD4^+^IL-17^+^ Th17 cells in both CHB and ASC were shown. **b** HBV core-specific CD4^+^CD25^+^CD127^dim/−^ Tregs percentage was comparable between CHB and ASC. **c** HBV core-specific CD4^+^IL-17^+^ Th17 cells percentage was significantly down-regulated in ASC when compared with CHB. **d** Ratio of Tregs/Th17 was notably increased in ASC when compared with CHB. Individual level for each subject was presented, and comparisons were made using Student *t* tests

PBMCs were isolated from all enrolled patients (21 CHB and 23 ASC), and were stimulated with HBc 18–27 peptide for 12 h. Cells were then stained with anti-CD4, −CD25, −CD127, and -IL-17 for flow cytometry analysis. The gating strategy and representative flow dots for Tregs (CD4^+^CD25^+^CD127^dim/−^) and Th17 (CD4^+^IL-17^+^) in both CHB and ASC was shown in Fig. 1a. There was no remarkable different of viral specific Tregs between CHB and ASC (11.99 ± 3.03% vs. 12.44 ± 2.84%, Student *t* test, *P* = 0.608, Fig. 1b). However, the percentage of viral specific Th17 cells in CHB was significantly higher than in ASC (0.97 ± 0.20% vs. 0.81 ± 0.13%, Student *t* test, *P* = 0.0024, Fig. 1c). Thus, ratio of HBc specific Tregs/Th17 in CHB was notably lower in comparison with ASC (12.62 ± 3.39 vs. 15.68 ± 4.27, Student *t* test, *P* = 0.012, Fig. 1d).

### IL-35 stimulation elevated HBV core-specific CD4^+^CD25^+^CD127^dim/−^ Tregs in CHB and ASC

**Fig. 2:**
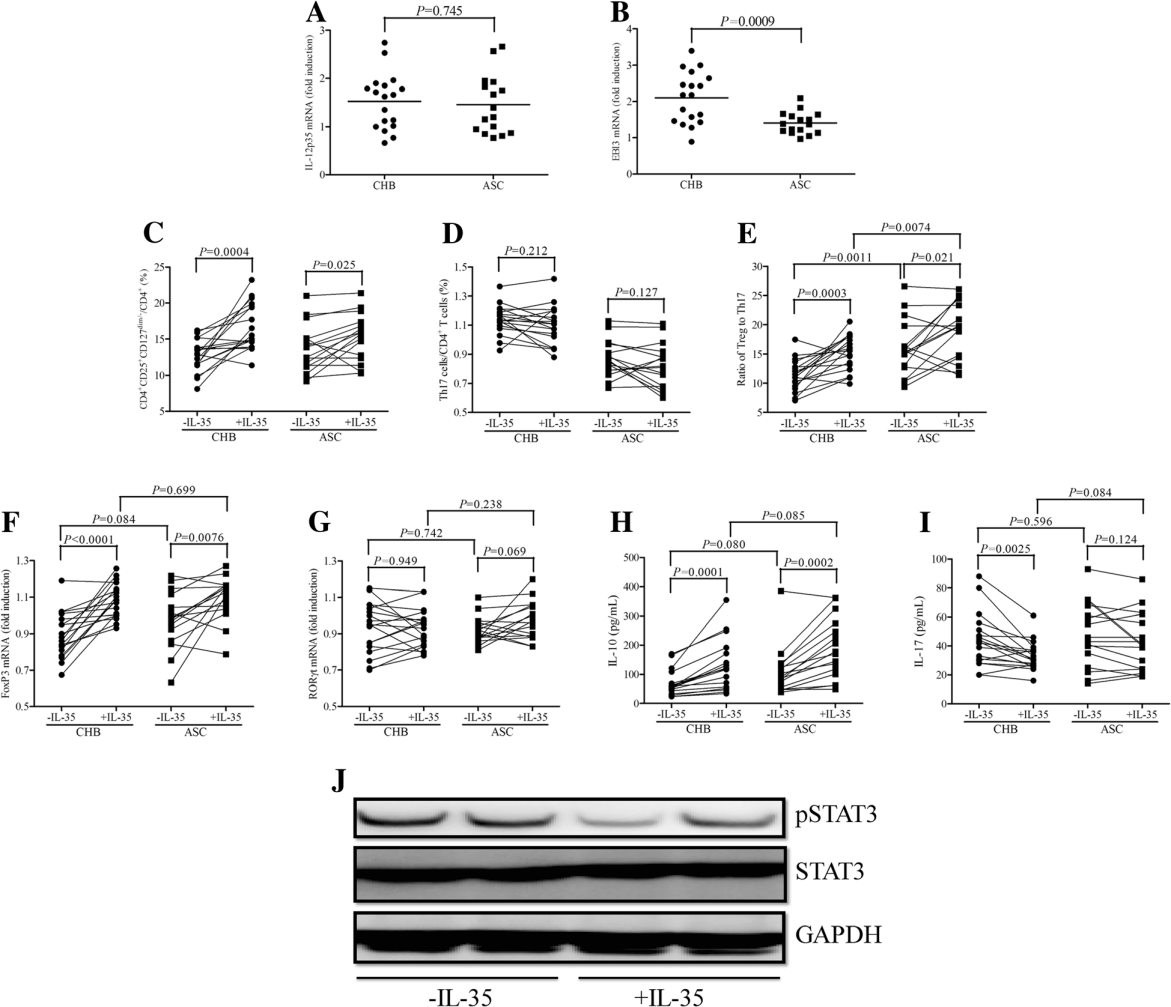
Interleukin (IL)-35 stimulation to HBV core-specific CD4^+^CD25^+^CD127^dim/−^ regulatory T cells (Tregs) and T helper 17 (Th17) cells in chronic hepatitis B (CHB) and asymptomatic HBV carriers (ASC). CD4^+^ T cells were purified from eighteen CHB and sixteen ASC, and were stimulated with or without IL-35 for 12 h in the presence of HBc 18–27 peptide. IL-12p35 and EBI3 mRNA expressions were investigated in unstimulated CD4^+^ T cells by real-time PCR. **a** IL-12p35 mRNA in CD4^+^ T cells was comparable between CHB and ASC. **b** EBI3 mRNA in CD4^+^ T cells was remarkably elevated in CD4^+^ T cells from CHB when compared with ASC. Individual level for each subject was presented, and comparisons were made using Student *t* tests. HBV core-specific CD4^+^CD25^+^CD127^dim/−^ Tregs and CD4^+^IL-17^+^ Th17 cells with or without IL-35 stimulation were investigated by flow cytometry. **c** HBV core-specific CD4^+^CD25^+^CD127^dim/−^ Tregs percentage was increased in response to IL-35 stimulation in both CHB and ASC. **d** HBV core-specific CD4^+^IL-17^+^ Th17 cells percentage was comparable between IL-35 stimulated and unstimulated CD4^+^ T cells in both CHB and ASC. **e** Ratio of Tregs/Th17 was increased in response to IL-35 stimulation in both CHB and ASC. Individual level for each subject was presented, and comparisons were made using paired *t* tests. mRNA relative expressions corresponding to Tregs transcriptional factor FoxP3 and Th17 transcriptional factor RORγt were measured by real-time PCR. **f** FoxP3 mRNA was notably increased in response to IL-35 stimulation in both CHB and ASC. **g** RORγt mRNA did not change significantly in response to IL-35 stimulation in either CHB or ASC. Individual level for each subject was presented, and comparisons were made using paired *t* tests. IL-10 and IL-17 production in cultured supernatants were measured by ELISA. **h** IL-10 expression in cultured supernatants was notably elevated in response to IL-35 stimulation in both CHB and ASC. **i** IL-17 expression in cultured supernatants was remarkably decreased in response to IL-35 stimulation only in CHB, but not in ASC. Individual level for each subject was presented, and comparisons were made using Wilcoxon matched pairs tests. **j** phosphorylated STAT3 (pSTAT3), STAT3, and GAPDH expression in CD4^+^ T cells with or without IL-35 stimulation was investigated by Western blot. pSTAT3 was significantly down-regulated in CD4^+^ T cells in response to IL-35 stimulation, however, total STAT3 expression was comparable in CD4^+^ T cells with or without IL-35 stimulation

CD4^+^ T cells were purified from eighteen CHB and sixteen ASC, and were stimulated with IL-35 for 12 h in the presence of HBc 18–27 peptide. Cells and supernatants were harvested for further experiments. Our previous study has been demonstrated serum IL-35 was elevated in both CHB and ASC [17]. It was also accepted that Tregs and other cell types (including activated myeloid, endothelial cells, regulatory B cells) could secret IL-35 [19]. Thus, IL-12p35 and EBI3 mRNA expression in unstimulated CD4^+^ T cells was firstly screened by real-time PCR. IL-12p35 mRNA was comparable between CHB and ASC (Student *t* test, *P* = 0.745, Fig. 2a). However, EBI3 mRNA was significantly elevated in CD4^+^ T cells from CHB when compared with those from ASC (Student *t* test, *P* = 0.0009, Fig. 2b). IL-35 stimulation notably increased CD4^+^CD25^+^CD127^dim/−^ Tregs percentage within CD4^+^ T cells in both CHB (16.68 ± 3.21% vs. 12.73 ± 2.24%, paired *t* test, *P* = 0.0004, Fig. 2c) and ASC (15.31 ± 3.20% vs. 13.64 ± 3.32%, paired *t* test, *P* = 0.025, Fig. 2c). However, IL-35 treatment only slightly down-regulated Th17 cells frequency in CHB (1.14 ± 0.10% vs. 1.10 ± 0.14%) and ASC (0.87 ± 0.12% vs. 0.82 ± 0.15%), and those differences failed to achieve significances (paired *t* tests, *P* = 0.212 and *P* = 0.127, respectively, Fig. 2d). Although ratio of Tregs/Th17 was remarkable elevated in both CHB (15.28 ± 2.98 vs. 11.35 ± 2.98, paired *t* test, *P* = 0.0003, Fig. 2e) and ASC (19.20 ± 4.87 vs. 16.03 ± 4.76, paired *t* test, *P* = 0.021, Fig. 2e) in response to IL-35 stimulation, this ratio was still higher in ASC in comparison with in CHB (Student *t* test, *P* = 0.0074, Fig. 2e). mRNA relative expression corresponding to key transcriptional factor for Tregs (FoxP3) and Th17 cells (RORγt) was investigated by real-time PCR. FoxP3 mRNA was elevated in both CHB (paired *t* test, *P* < 0.0001, Fig. 2f) and ASC (paired *t* test, *P* = 0.0076, Fig. 2f) in response IL-35 stimulation. However, IL-35 treatment did not affect RORγt mRNA expression in CD4^+^ T cells from either CHB (paired *t* test, *P* = 0.949, Fig. 2g) or ASC (paired *t* test, *P* = 0.069, Fig. 2g). Tregs-secreting IL-10 and Th17-secreting IL-17 production in the cultured supernatants were also measured by ELISA. IL-10 expression was elevated in response to IL-35 stimulation in CHB (131.4[55.52, 177.3] vs. 68.61[41.13, 79.40], Wilcoxon matched pairs test, *P* = 0.0001, Fig. 2h) and ASC (189.3[105.3, 268.6] vs. 109.3[50.35, 130.0], Wilcoxon matched pairs test, *P* = 0.0002, Fig. 2h). IL-17 production by CD4^+^ T cells was down-regulated with IL-35 stimulation in CHB (33.11[26.09, 37.79] vs. 43.78[29.69, 52.06], Wilcoxon matched pairs test, *P* = 0.0025, Fig. 2i), however, was comparable in ASC in presence or absence of IL-35 treatment (42.69[24.12, 60.03] vs. 44.56[27.45, 66.61], Wilcoxon matched pairs test, *P* = 0.124, Fig. 2i). pSTAT3 and STAT3 expression in CD4^+^ T cells was investigated by Western blot (Fig. 2j). STAT3 phosphorylation was significantly down-regulated in CD4^+^ T cells in response to IL-35 treatment, however, total STAT3 expression was comparable in the presence or absence of IL-35 stimulation (Fig. 2j).

### IL-35 stimulation reduced viral specific Th17 differentiation of Tregs in CHB

**Fig. 3:**
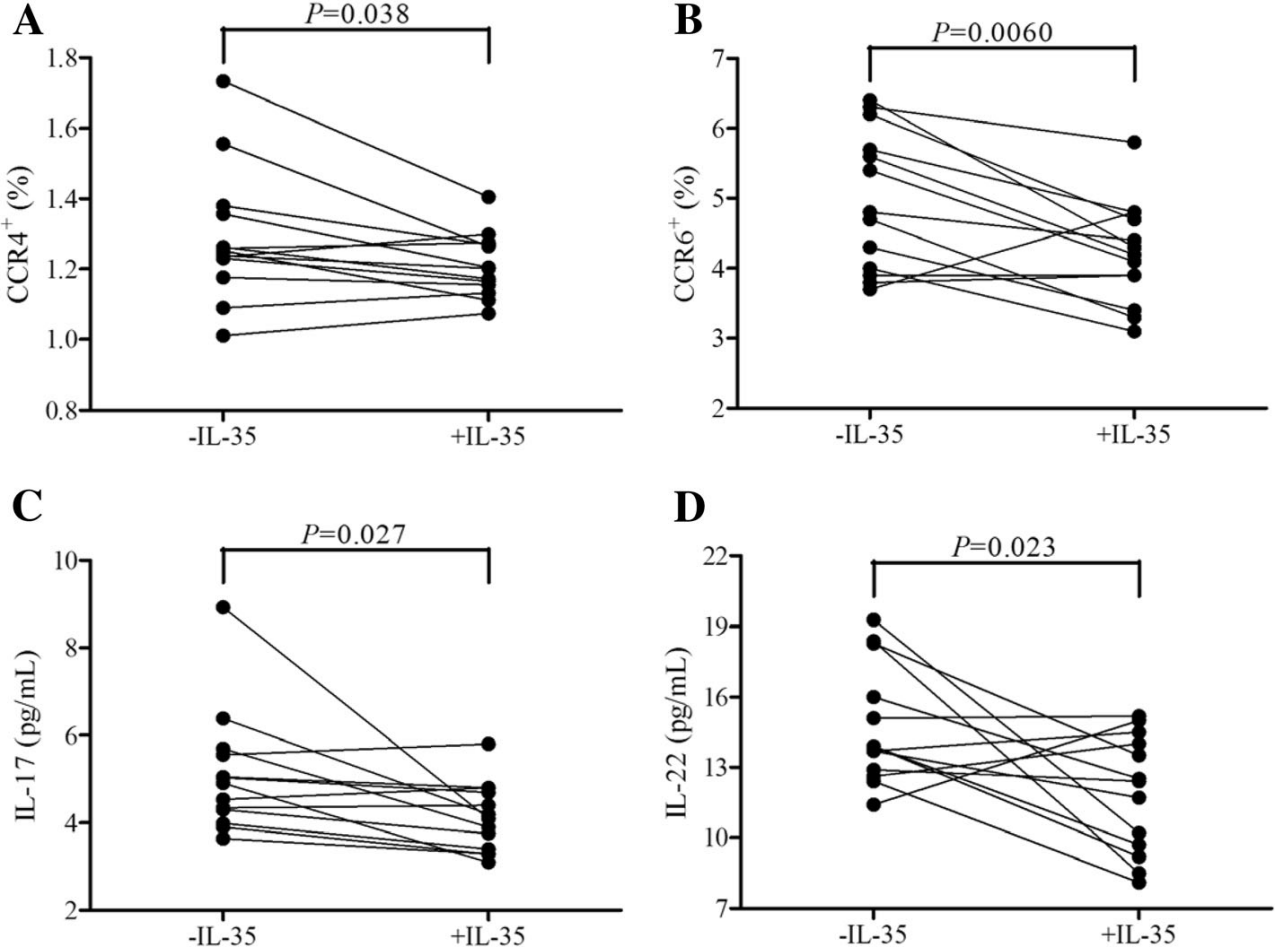
T helper 17 (Th17)-like phenotype differentiation from CD4^+^CD25^+^CD127^dim/−^ regulatory T cells (Tregs) in chronic hepatitis B (CHB) in response to interleukin (IL)-35 stimulation. CD4^+^CD25^+^CD127^dim/−^ Tregs were purified from thirteen CHB patients, and were stimulated with HBc 18–17 peptide in the presence or absence of IL-35 for 12 h. CCR4^+^ and CCR6^+^ cells in purified Tregs were investigated by flow cytometry. **a** CCR4^+^ Trges percentage was significantly down-regulated in response to IL-35 stimulation. **b** CCR6^+^ Trges percentage was significantly down-regulated in response to IL-35 stimulation. IL-17 and IL-22 production in cultured supernatants were measured by ELISA. **c** IL-17 expression in cultured supernatants was decreased in response to IL-35 stimulation. **d** IL-22 expression in cultured supernatants was decreased in response to IL-35 stimulation. Individual level for each subject was presented, and comparisons were made using paired *t* tests

Our previous study has been demonstrated that IL-35 stimulation in vitro robustly promoted inhibitory activity of CD4^+^CD25^+^CD127^dim/−^ Tregs [17]. Th17-like phenotype could also be induced from both human naïve and effector Tregs, leading to reduced suppressive function [20, 21]. Thus, we purified CD4^+^CD25^+^CD127^dim/−^ Tregs from thirteen CHB patients, and were stimulated with HBc 18–17 peptide in the presence or absence of IL-35 for 12 h. Cells were harvested and tested for CCR4 and CCR6 expression by flow cytometry. Both CCR4^+^ (1.21 ± 0.09% vs. 1.29 ± 0.19%, paired *t* test, *P* = 0.038, Fig. 3a) and CCR6^+^ percentage (4.21 ± 0.73% vs. 4.99 ± 1.00%, paired *t* test, *P* = 0.006, Fig. 3b) within Tregs was significantly reduced in response to IL-35 stimulation. IL-17 and IL-22 production by Tregs was also measured in cultured supernatants. Both IL-17 (4.12 ± 0.78 pg/mL vs. 5.10 ± 1.39 pg/mL, paired *t* test, *P* = 0.027, Fig. 3c) and IL-22 secretion (11.88 ± 2.52 pg/mL vs. 14.74 ± 2.53 pg/mL, paired *t* test, *P* = 0.023, Fig. 3d) was remarkably down-regulated with IL-35 stimulation.

### Effective antiviral therapy reduced responsiveness of CD4^+^ T cells and Tregs to IL-35 stimulation in CHB

**Fig. 4:**
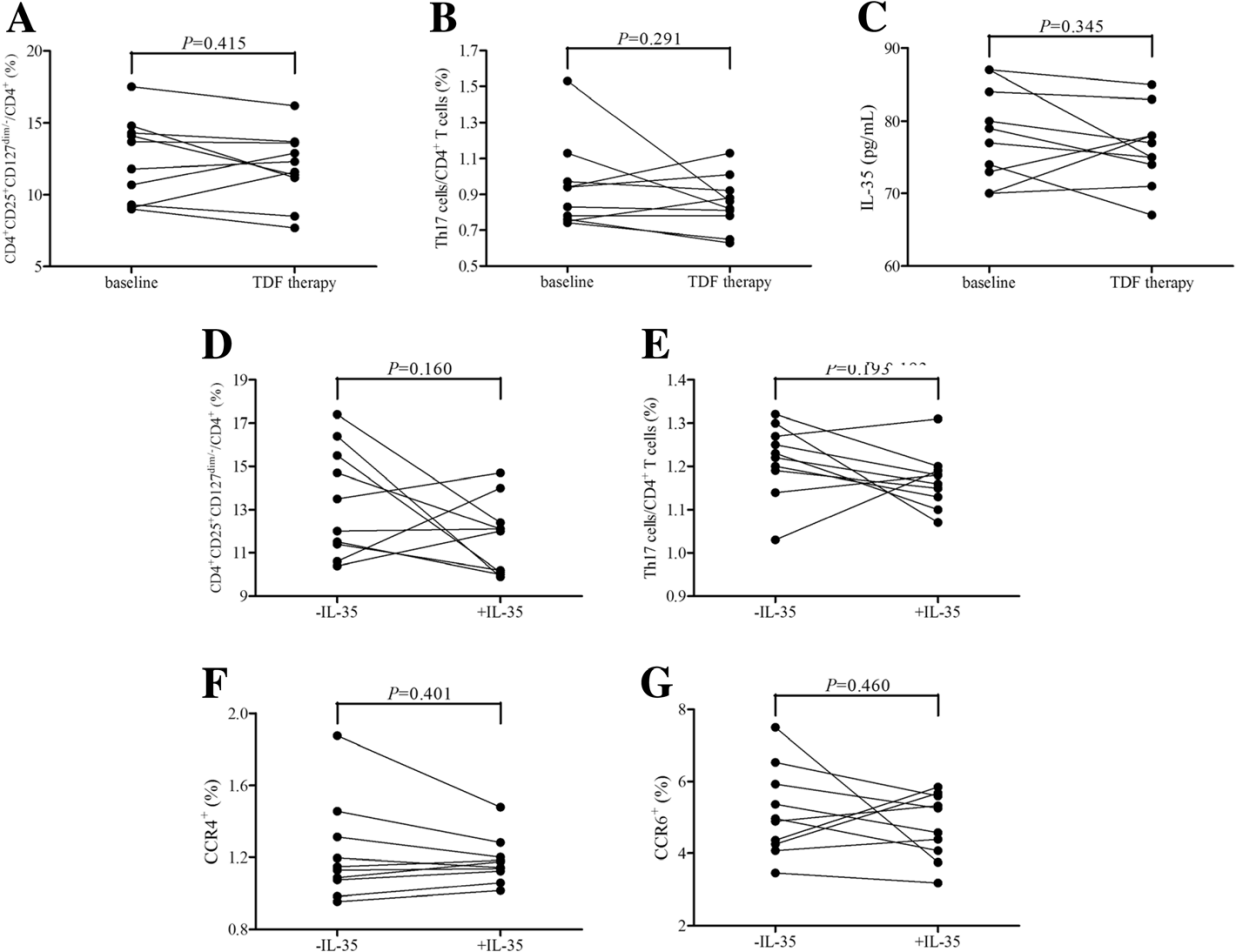
Influence of effective antiviral therapy to HBV core specific CD4^+^CD25^+^CD127^dim/−^ regulatory T cells (Tregs), T helper 17 (Th17) cells, and responsiveness to interleukin (IL)-35 stimulation. Ten of chronic hepatitis B (CHB) patients received tenofovir disoproxil fumarate (TDF, 300 mg once daily). Peripheral blood mononuclear cells (PBMCs), CD4^+^ T cells, and CD4^+^CD25^+^CD127^dim/−^ Tregs were isolated and purified three months post-therapy when reaching virological response. HBV core-specific CD4^+^CD25^+^CD127^dim/−^ Tregs and CD4^+^IL-17^+^ Th17 cells prior to and post TDF therapy were investigated by flow cytometry, and IL-35 expression in the serum was measured by ELISA. **a** CD4^+^CD25^+^CD127^dim/−^ Tregs percentage, (**b**) CD4^+^IL-17^+^ Th17 cells percentage, and (**c**) IL-35 expression did not change significantly in response to TDF-therapy. Purified CD4^+^ T cells from TDF-treated CHB patients were stimulated with HBc 18–17 peptide in the presence or absence of IL-35 for 12 h. HBV core-specific CD4^+^CD25^+^CD127^dim/−^ Tregs and CD4^+^IL-17^+^ Th17 cells were investigated by flow cytometry. **d** CD4^+^CD25^+^CD127^dim/−^ Tregs and (**e**) CD4^+^IL-17^+^ Th17 cells percentage in purified CD4^+^ T cells from TDF-treated patients did not change significantly in response to IL-35 stimulation. Purified CD4^+^CD25^+^CD127^dim/−^ Tregs from TDF-treated CHB patients were stimulated with HBc 18–17 peptide in the presence or absence of IL-35 for 12 h. **f** CCR4^+^ cells and (**g**) CCR6^+^ cells in purified Tregs from TDF-treated patients did not change significantly in response to IL-35 stimulation. Individual level for each subject was presented, and comparisons were made using paired *t* tests

Ten of CHB patients received tenofovir disoproxil fumarate (TDF, 300 mg once daily), and all ten patients reached virological response with undetectable HBV DNA in the serum three month post-therapy. CD4^+^ T cells and CD4^+^CD25^+^CD127^dim/−^ Tregs were purified, and were stimulated with IL-35 in the presence of HBc 18–27 peptide for 12 h. There were no significant difference in viral specific Tregs (11.91 ± 2.49% vs. 12.43 ± 2.89%, paired *t* test, *P* = 0.415, Fig. 4a), Th17 cells (0.85 ± 0.15% vs. 0.94 ± 0.24%, paired *t* test, *P* = 0.291, Fig. 4b), or IL-35 expression in the serum (76.30 ± 5.27 pg/mL vs. 78.10 ± 6.44 pg/mL, paired *t* test, *P* = 0.345, Fig. 4c) in response to TDF therapy. IL-35 stimulation in vitro did not elevated viral specific Tregs (11.75 ± 1.70% vs. 13.34 ± 2.53%, paired *t* test, *P* = 0.160, Fig. 4d) or Th17 cells (1.17 ± 0.06% vs. 1.22 ± 0.08%, paired *t* test, *P* = 0.193, Fig. 4e) within CD4^+^ T cells from TDF-treated CHB patients. Moreover, CCR4^+^ (1.18 ± 0.13% vs. 1.22 ± 0.27%, paired *t* test, *P* = 0.401, Fig. 4f) and CCR6^+^ (4.77 ± 0.91% vs. 5.13 ± 1.23%, paired *t* test, *P* = 0.460, Fig. 4g) cells were also did not affected by IL-35 in vitro stimulation in purified CD4^+^CD25^+^CD127^dim/−^ Tregs from TDF-treated CHB patients.

## Discussion

In the present study, an increased HBV core-specific Th17 cell response was found in CHB patients, leading to the reduction of viral specific Tregs/Th17 ratio in CHB patients when compared with ASC. Recombinant human IL-35 stimulation elevated HBV core-specific CD4^+^CD25^+^CD127^dim/−^ Tregs frequency, FoxP3 mRNA relative level, and IL-10 production in both CHB and ASC. However, there was no remarkable differences of viral specific Th17 cells and RORγt mRNA in response to IL-35 stimulation. More importantly, IL-35 stimulation reduced HBV core-specific Th17 differentiation from Tregs in CHB. Effective antiviral therapy did not affect viral specific Tregs/Th17 cells frequency or IL-35 expression in CHB patients, but downregulated responsiveness of CD4^+^ T cells or CD4^+^CD25^+^CD127^dim/−^ Tregs to IL-35 stimulation in vitro. The current results indicated a potential mechanism of IL-35-induced immunoregulation in chronic HBV infection.

Chronic HBV infection led to the imbalance between Tregs and Th17 cells, which mainly presented as the changes of percentage and function of both cell subsets in different disease stages. Imbalance of Tregs and Th17 cells played an important role in the occurrence, development and outcome of CHB [12, 22]. Feng et al. demonstrated both elevation of Tregs and Th17 cells, however, reduced ratio of Tregs/Th17 cells in chronic HBV infection, which were independent risk factors to hepatitis [23]. In contrast, some other studies reported that Treg cell levels decreased, while Th17 cell levels increased in peripheral blood of CHB patients [24, 25]. Tregs/Th17 imbalance was also associated with the survival and progression in HBV-associated liver cirrhosis [26, 27] and in acute-on-chronic hepatitis B liver failure [28, 29]. The imbalance between Tregs and Th17 cells served as a risk factor for HCC occurrence following cirrhosis [13, 30]. Restoring the Tregs to Th17 cell ratio might maintained immune system at a steady state, which alleviated liver injury in HBV-related acute-on-chronic liver failure [31]. However, few studies focused on viral specific Tregs/Th17 cells imbalance during HBV infection. To the best of our knowledge, we firstly revealed an imbalance of HBV core-specific CD4^+^CD25^+^CD127^dim/−^ Tregs and Th17 cells in CHB patients, which presented as increased viral specific Th17 frequency but comparable viral specific Tregs percentage between CHB and ASC. Moreover, previous reports also indicated sustained changes of Tregs/Th17 cells and their related cytokines were closely related to virological, biochemical, and serological responses to nucleos(t)ide analogues (including entecavir, lamivudine, adefovir, and telbivudine) [32–34] and interferon-α therapy [35]. However, we found that effective anti-HBV therapy by TDF did not restore the imbalance between HBV core-specific Tregs and Th17 cells three months post initiation of treatment. This might be partly due to the relatively short followed-up period. Further experiments were needed for investigating the changes of viral specific Tregs/Th17 imbalance in CHB patients with long-term administration of anti-HBV drugs or with HBeAg seroconversion.

IL-35 functioned as an immunosuppressive factor in immune-mediated diseases. The predominant mechanism of suppression was to inhibit proliferation and effector activities of T cells, thus, might be a potential therapeutic target for controlling HBV infection [19]. Both serum IL-35 and IL-35 mRNA in CD4^+^ T cells was elevated in chronic HBV infection, and was associated with viral replication and hepatocytes damage [36, 37]. Recombinant IL-35 stimulation not only directly increased HBV transcription and replication in vivo [38], but also suppressed HBV core-specific interferon-γ-secreting CD8^+^ T cells in vitro [39]. Our previous study also indicated an immunosuppressive activity of IL-35 in chronic HBV infection [17]. The elevated IL-35 in CHB and ASC enhanced suppressive function of CD4^+^CD25^+^CD127^dim/−^ Tregs, while reduced cytolytic and noncytolytic activity of HBV antigen-specific CD8^+^ T cells, which might due to the direct response and positive feedback mechanisms of Tregs to IL-35 [17]. IL-35 was shown to mitigate the function of murine transplanted islet cells via regulation of the Treg/Th17 ratio [40]. In enterovirus 71-induced hand, foot, and mouth disease, recombinant IL-35 directly modulated Tregs/Th17 imbalance [18]. The current results revealed that IL-35 reduced the phosphorylation of STAT3 in cultured CD4^+^ T cells, suggesting the activation of IL-35-induced signaling pathways. IL-35 stimulation mainly affect viral specific CD4^+^CD25^+^CD127^dim/−^ Tregs, which presented as the elevation of cellular frequency, FoxP3 mRNA, and IL-10 production within CD4^+^ T cells from both CHB and ASC. However, Th17 cells frequency, RORγt mRNA, or IL-17 secretion by CD4^+^ T cells did not change notably in response to IL-35 stimulation. This was not consistent with the previous results in coxsackievirus-B3-induced myocarditis [41] and hemocytopenia [42], in which Th17 cells was strongly inhibited by delivery of IL-35. This might be partly due to the difference status of chronic and acute viral infections. Moreover, we also found that IL-35 treatment partly restored the imbalance of HBV core-specific Tregs and Th17 cells in CHB patients, however, ratio of viral specific Tregs/Th17 cells in CHB patients was still lower than that in ASC in response to IL-35 stimulation.

Antigen stimulation and/or pathogen infections could induce IL-17 production by CD4^+^FoxP3^+^ Tregs [43]. The CD4^+^RORγt^+^FoxP3^+^ T cells represented intermediates during Tregs/Th17 transdifferentiation, and constituted an independent biofunctional regulatory cell lineage which contributed to cresecentic glomerulonephritis [44]. Toll-like receptor 2 (TLR2) signaling promoted IL-17 production in Tregs during oropharyngeal candidiasis [45] and HCV infection [21]. The current data revealed that IL-35 stimulation inhibited the differentiation of HBV core-specific Tregs into a Th17-like phenotype by dampening IL-17/IL-22 production and reducing CCR4/CCR6 expression. Furthermore, effective inhibition of HBV replication by TDF induced the decreased CD4^+^CD25^+^CD127^dim/−^ Tregs and Th17 cell responsiveness to IL-35 in CHB patients. This was consistent with the reduced responsiveness of Tregs/Th17 to TLR2 in chronic HCV infected patients with direct-acting antiviral drugs therapy [46]. This might imply that chronic HBV infection might increase the responsiveness of CD4^+^CD25^+^CD127^dim/−^ Tregs to IL-35, and in turn contribute to elevated suppressive activity and to viral persistence. Thus, elevated IL-35 promoted the CD4^+^CD25^+^CD127^dim/−^ Tregs frequency and reduced Th17 differentiation of Tregs, leading to the Treg/Th17 imbalance and contributing to HBV persistence.

There were limitations of the current study. Firstly, the experiments were solely performed on PBMC due to the limited HLA-A2 restricted patients enrolled. A large number of cases could be analyzed in order to obtained a suitable statistic number to compare. Secondly, cells in the peripheral blood do not accurately represented the intra-hepatic environment. Thus, further in vivo experiments or purified lymphocytes from liver biopsy samples were needed to confirm the in vitro results.

## Conclusion

In summary, we demonstrated that IL-35 modulated viral specific Tregs/Th17 cell balance in patients with chronic HBV infection. Inhibition of HBV replication led to reduction in the responsiveness of IL-17-secreting phenotypic shift of Tregs upon IL-35 stimulation. Such novel mechanisms might enable deliberate manipulation targeting viral specific Tregs/Th17 transdifferentiation by IL-35 for therapeutic strategy of chronic HBV infection.

## Abbreviations

ALT: Alanine aminotransferase
ASC: Asymptomatic HBV carriers
CHB: Chronic hepatitis B
EBI3: Epstein-Barr virus-induced gene 3
ELISA: Enzyme linked immunosorbent assay
HBcAg: Hepatitis B core antigen
HBeAg: HBV e antigen
HBsAg: HBV surface antigen
HBV: Hepatitis B virus
HCC: Hepatocellular carcinoma
HCV: Hepatitis C virus
HIV: Human immunodeficiency virus
IL: Interleukin
PBMC: Peripheral blood mononuclear cells
RORγt: Retinoic acid-related orphan receptor γt
STAT3: Signal transducers and activators of transcription 3
Th: T helper
Tregs: Regulatory T cells
